# Effectiveness and cost‐effectiveness of face‐to‐face and electronic brief interventions versus screening alone to reduce alcohol consumption among high‐risk adolescents presenting to emergency departments: three‐arm pragmatic randomized trial (SIPS Junior high risk trial)

**DOI:** 10.1111/add.15884

**Published:** 2022-04-12

**Authors:** Paolo Deluca, Simon Coulton, Mohammed Fasihul Alam, Sadie Boniface, Kim Donoghue, Eilish Gilvarry, Eileen Kaner, Ellen Lynch, Ian Maconochie, Paul McArdle, Ruth McGovern, Dorothy Newbury‐Birch, Robert Patton, Tracy Pellat‐Higgins, Ceri Phillips, Thomas Phillips, Rhys D. Pockett, Ian T. Russell, John Strang, Colin Drummond

**Affiliations:** ^1^ Addictions Department National Addiction Centre, Institute of Psychiatry, Psychology and Neuroscience, King's College London London UK; ^2^ Centre for Health Services Studies University of Kent Canterbury UK; ^3^ Department of Public Health, College of Health Sciences, QU Health Qatar University Qatar; ^4^ Institute of Alcohol Studies Alliance House London UK; ^5^ Department of Clinical, Educational and Health Psychology University College London London UK; ^6^ Northumberland Tyne and Wear NHS Foundation Trust, St Nicholas Hospital Newcastle upon Tyne UK; ^7^ Institute of Health and Society, Baddiley‐Clark Building Newcastle University Newcastle upon Tyne UK; ^8^ Paediatric Emergency Medicine Imperial College London, Queen Elizabeth the Queen Mother Wing, St Mary's Hospital London UK; ^9^ School of Social Sciences, Humanities and Law Teesside University Middlesbrough UK; ^10^ School of Psychology, Elizabeth Fry Building (AD) University of Surrey Guildford UK; ^11^ Medical School Swansea University Swansea UK; ^12^ Institute for Clinical and Applied Health Research University of Hull Hull UK; ^13^ Swansea Centre for Health Economics, College of Human and Health Sciences Swansea University Swansea UK; ^14^ South London and Maudsley NHS Foundation Trust London UK

**Keywords:** Adolescent, alcohol, alcohol screening, brief intervention, cost‐effectiveness, effectiveness, electronic brief intervention, emergency department, high risk, pragmatic randomized trial

## Abstract

**Background and aims:**

Alcohol use increases throughout adolescence. Emergency department (ED) attendance is an opportunity for alcohol screening and brief intervention (ASBI), which is effective for adults. This trial evaluated the effectiveness and cost‐effectiveness of ASBI compared with screening alone (SA) in high‐risk adolescents.

**Design, Setting and Participants:**

Multi‐centre, three‐group, single‐blind, individually randomized trial with follow‐ups after 6 and 12 months in 10 ED settings in England. From October 2014 to May 2015 we screened 3327 adolescents aged 14 to 18 years, of whom 756 (22.7%) scored at least 3 on the Alcohol Use Disorders Identification Test: consumption (AUDIT‐C) and consented to participate in this trial. Mean age was 16.1 years; 50.2% were female and 84.9% were white.

**Interventions:**

Interventions were personalized feedback and brief advice (PFBA), personalized feedback plus electronic brief intervention (eBI) and SA.

**Measures:**

The primary outcome was the weekly alcohol consumed in standard UK units (8 g ethanol) at 12 months post‐randomization, derived from extended AUDIT‐C. Economic outcomes included quality of life and service use, from perspectives of both the National Health Service and personal social services (NHS&PSS) and society.

**Findings:**

At 12 months, mean weekly consumption was 2.99 [95% confidence interval (CI) = 2.38–3.70] standard units for the SA group, 3.56 (95% CI = 2.90, 4.32) for PFBA and 3.18 (95% CI = 2.50, 3.97) for eBI, showing no significant differences. The PFBA group consumed mean 0.57 (−0.36, 1.70) units more than SA; and eBIs consumed 0.19 (−0.71, 1.30) more. Bayes factors suggested lack of effectiveness explained non‐significance. From the NHS&PSS perspective, economic analysis showed that PFBA and eBI were not cost‐effective compared with SA: PFBA yielded incremental cost‐effectiveness ratio of £6213 (−£736 843, £812 884), with the intervention having 54% probability of being cost‐effective compared with SA at the £20 000 WTP threshold.

**Conclusions:**

In emergency departments in England, neither personalized feedback and brief advice nor personalized feedback plus electronic brief intervention showed evidence of being effective or cost‐effective when compared with screening alone in reducing alcohol consumption among adolescents.

## INTRODUCTION

Alcohol is a global public health problem and a major health concern in adolescence. A European survey found that 80% of 14‐ and 15‐year‐olds across 35 countries had consumed alcohol, and 48% had done so more than once during the past month [1]. While alcohol consumption has fallen in recent years among young people, the 2018 Smoking Drinking and Drug Use survey of 11–15‐year‐old schoolchildren estimated that 306 000 adolescents in England had drunk alcohol during the last week [2]. There is also evidence from previous surveys of increases in the mean amount consumed by those who drank alcohol [3]. In 2018, pupils who drank alcohol during the last week had consumed an average of 10.3 units that week (up from 6.4 in 1994); 21% of them were estimated to have drunk more than 15 units [2].

Alcohol use rises steeply throughout adolescence [4]. Excessive alcohol consumption in adolescence increases the risk of unprotected or regretted sexual activity, disorderly or criminal behaviour, self‐harm and suicide, accidents and injuries, alcohol poisoning and accidental death [5]. Adolescent alcohol consumption is linked to alcohol problems later in life, including dependence, physical and mental ill‐health and social consequences [6].

Alcohol screening and brief intervention (ASBI) has strong evidence in adults in both primary care and emergency departments (EDs) of reducing alcohol consumption in hazardous and harmful drinkers compared with minimal or no intervention [7, 8]. While ASBI encompasses a wide range of approaches, it is generally an opportunistic intervention during clinical consultation. EDs can potentially exploit alcohol‐related attendance, as patients may be more receptive to advice about their drinking. There is evidence of effectiveness and cost‐effectiveness of ASBI in EDs in adults [9, 10]. Among adolescents, most of the evidence about ASBI is taken from educational settings. There has been much less research on ASBI for adolescents in health‐care settings, but a few trials have reported reduced alcohol consumption [11, 12, 13].

Recent systematic reviews suggest that ASBI delivered via the internet can significantly reduce alcohol consumption in adults compared with minimal or no intervention [14]. Electronic brief interventions can be web‐based or smartphone applications (apps) and have advantages of acceptability, anonymity and scalability compared with clinician‐delivered ASBI [14]. The rise in smartphone ownership opens the possibility of wide implementation of eBI. However, there are few published studies of these interventions delivered by smartphones or by targeting adolescents.

We conducted a randomized trial in adolescents identified as drinkers at high‐risk attending EDs in England. We compared screening alone (SA) with two forms of ASBI: face‐to‐face personalized feedback and brief advice (PFBA) and personalized feedback and electronic brief intervention (eBI). We complemented this with another randomized trial of the same interventions in abstinent or low‐risk drinkers recruited in the same setting, reported separately [15]. This research forms part of the Screening and Intervention Programme for Sensible drinking (SIPS) Junior research programme.

The trial aimed to compare these two forms of ASBI with SA in hazardously drinking adolescents attending ED to evaluate their effectiveness and cost‐effectiveness in reducing alcohol consumption (primary outcome) and the Alcohol Use Disorders Identification Test: consumption (AUDIT‐C) score (secondary outcome), and to identify prognostic and psychological factors which predict changes in drinking behaviour. The null hypothesis was that PFBA and eBI are not effective or cost‐effective compared to SA in reducing alcohol consumption 12 months after randomization.

## METHODS

### Design

The design comprised a multi‐centre, single‐blind, pragmatic, individually randomized trial with three parallel groups comparing PFBA, eBI and SA in adolescents drinking at high risk and following them up at 6 and 12 months after randomization; the trial protocol has been published [16].

### Setting

We undertook the trial in 10 EDs across three regions of England (North East, Yorkshire and Humber and London). We recruited participants between 1000 and 2200 hours, 7 days per week between October 2014 and May 2015. Trained researchers interviewed consecutive ED attenders between their 14th and 18th birthdays following clearance from ED clinical staff that they were well enough to participate and had given consent to be approached. We trained these researchers in all trial procedures and in delivering the interventions, notably through demonstrations and role‐play. All were experienced in addictions care or research or both.

### Participants

#### Inclusion criteria

Inclusion criteria were ED attenders between their 14th and 18th birthdays who: scored ≥ 3 on the Alcohol Use Disorders Identification Test: consumption (AUDIT‐C) questionnaire [17, 18]; were alert and orientated; could speak English sufficiently well to complete the research assessment; resided within 20 miles of the ED; were able and willing to provide informed consent to screening, intervention and follow‐up; if under 16 years, were ‘Gillick competent’ [19] or whose parent or guardian provided informed consent; and had a smartphone or access to the internet at home. Those scoring < 3 on AUDIT‐C were eligible for the parallel low‐risk trial [15].

#### Exclusion criteria

Exclusion criteria included severe injury; gross intoxication; under care of specialist services for social or psychological needs; in receipt of treatment for alcohol or substance use within 6 months; participating in another alcohol‐related research study.

### Ethics statement

We conducted the trial in accordance with the Declaration of Helsinki. We received full NHS ethics approval (reference 14/LO/0721). We registered it with the International Standard Randomized Controlled Trials Number Registry as ISRCTN 45300218. We obtained Research and Development approval from all participating NHS organizations. To reduce the burden on participants, and with the agreement of the Trial Steering Committee we modified the published trial protocol [16] to assess consumption by AUDIT‐C at follow‐up rather than the more complex time‐line follow‐back (TLFB) 28‐day method [20].

### Consent

Once ED clinical staff had cleared potential participants to be approached, a researcher introduced the trial to them in a private area of the ED, and their parent or guardian if they were aged < 16 years. Researchers described the study as being about alcohol, life‐style and health, focusing upon preventing alcohol‐related harm in young people irrespective of their alcohol consumption, as there was a parallel trial for those at low risk, including abstainers. They explained it both orally and by giving them the patient information sheet (also given to the parent or guardian if the potential participant was aged < 16 years and accompanied). Potential participants, and parents or guardians where applicable, had up to 4 hours to ask questions about the study and decide whether to take part. Researchers used an electronic tablet (iPad) to check eligibility for the trial. They invited eligible participants, and parents or guardians where applicable, to give informed consent, including permission to access their ED records and agreement to participate in the interventions and follow‐up after 6 and 12 months.

### Screening and baseline assessment

Once consented, the participant took approximately 10 minutes to complete the alcohol screening and baseline questionnaire on the iPad, supervised by the researcher. The questionnaire included: demographic information; health and life‐style questions; the AUDIT‐C questionnaire [18]; items 19, 21 and 22 of the European School Project on Alcohol and other Drugs (ESPAD) [21]; the strengths and difficulties questionnaire [22]; the EQ‐5D‐5L (EuroQol, five dimensions–five levels) [23]; and a short service use questionnaire. We then allocated participants scoring ≥ 3 on AUDIT‐C [17] at random to one of the three interventions. Trained researchers delivered the allocated intervention, thanked participants for taking part, gave them £5 vouchers and returned them to the care of ED staff.

### Randomization and masking

Randomization employed random permuted blocks of varying size stratified by ED and gender and a participant had an equal probability of allocation to any of the three groups. Randomization strings were generated by a secure, independent randomization service and only released at the point of randomization through the iPad.

It was not possible or desirable to blind participants or interventionists to the allocated interventions. We also blinded researchers conducting follow‐up at 6 and 12 months, and those undertaking analysis.

### Interventions and comparator

Table 1 briefly summarizes the components of the comparator and the two active interventions.

**TABLE 1:** Summary of trial arm components

Component	Screening alone (SA)	Personalized feedback and brief advice (PFBA)	Personalized feedback and electronic brief intervention (eBI)
Rational, theory or goal	Control condition	Brief advice to achieve abstinence or low‐level consumption	Brief advice delivered via interactive electronic app. to achieve abstinence or low‐level consumption
Materials	None	Healthy Lifestyle leaflet	Healthy Lifestyle leaflet and smartphone app.
Procedure	Screening only using AUDIT‐C	Personalized feedback on alcohol screening, and brief advice and discussion of alcohol use, covering feedback of screening result, recommended consumption levels, normalized consumption for age, strategies to achieve abstinence or low‐level drinking and sources of additional support	In addition to personalized feedback on their alcohol screening participants were introduced to a smartphone or PC‐based app. designed to help achieve abstinence or low‐level consumption. The app. centred around a city with a specific building where advice could be sought. Participants could create drinking diaries, create goals, receive personalized feedback and seek advice regarding risks associated with alcohol use
Interventionist	ED nurse or researcher	ED nurse or researcher	ED nurse or researcher, app. was self‐directed
Delivery mode	Screening tool self‐completed on iPAD	Face‐to‐face discussion	Interaction with app. was self‐directed
Location	Emergency department	Emergency department	Personalized feedback and initial introduction to the app was in the emergency department, interaction with the app. was at the participant's discretion
Session duration and frequency	1 minute, one occasion	Up to 5 minutes, one occasion	Personalized feedback and introduction to app. up to 20 minutes on one occasion. Interaction with the app. was not limited in terms of duration or frequency

ED = emergency department; AUDIT‐C = Alcohol Use Disorders Identification Test: consumption; app. = application.

#### Screening alone (SA)

After completing the baseline assessment, we thanked SA participants for taking part and reminded them that a researcher would contact them after 6 and 12 months for follow‐up.

#### Personalized feedback and brief advice (PFBA)

A trained researcher took 5 minutes to deliver structured alcohol advice. We adapted the SIPS brief advice about alcohol risk intervention to this high‐risk target population [16]. PFBA includes the following advice: recommended levels of alcohol consumption for young people (based on the UK Chief Medical Officer's guidance); summary of their screening results and their meaning; normative feedback on how the participant's drinking compares with other young people in England; risks of drinking and benefits of stopping or reducing alcohol consumption; strategies to help stop or reduce drinking; drinking goals to consider; and local information on where to obtain further help or support with drinking. The researcher then gave the participant a summary of this information to take home.

#### Personalized feedback plus electronic brief intervention (eBI)

We designed the ‘SIPS City’ off‐line‐capable web app. to work on both iPhone and Android OS phones. We developed it through co‐production with young people. It uses the concept of game‐playing, in which users explore, navigate, learn facts about alcohol, record alcohol consumption, receive personalized feedback and set goals in an engaging city‐scape format with the aim of supporting users to reduce or stop alcohol consumption. Researchers helped participants with smartphones to download the app. before leaving the ED and demonstrated its key features. For participants without access to a smartphone but with access to the internet through other computerized devices, researchers provided access to a web‐based version of the app. with instructions for use.

At the end of both active interventions, we thanked participants for taking part, and reminded them that a researcher would contact them after 6 and 12 months to conduct follow‐up interviews.

### Outcome measures

We planned to follow‐up all participants at 6 months after randomization with a brief questionnaire and again at 12 months with a full assessment. We conducted these interviews over the telephone, face‐to‐face or electronically via the internet, as preferred by the participant. We trained researchers to administer these assessments while remaining blind to the group allocation of participants. We sent all participants who completed 6‐ and 12‐month assessments a letter of thanks with another £5 gift token in recognition of their participation.

#### Primary outcome measure

The primary outcome measure was alcohol consumption at 12 months, derived from the extended‐item AUDIT‐C questionnaire. We originally planned to use the TLFB28 as the primary outcome measure; however, this cannot be easily administered over the internet in a self‐completion format, as it was designed for completion by a trained interviewer. Validation of the AUDIT‐C during our earlier ED alcohol screening study showed excellent levels of agreement between alcohol consumption derived from the extended AUDIT‐C and the TLFB28 [17], findings replicated by other studies comparing different methods of eliciting alcohol consumption [24, 25, 26]. Furthermore, several large randomized controlled trials (RCTs) have used AUDIT‐C as a primary outcome measure [27, 28, 29]. AUDIT‐C also shows good responsiveness to changes in alcohol consumption [30]. The extended AUDIT‐C enhances the responses for question 1 (frequency of consumption), by replacing ‘four or more times per week’ with ‘four to five times per week’ and ‘six or more times per week’; and for question 2 (mean quantity consumed) by replacing ‘10 or more standard drinks’ with three new categories, ‘10 to 11’, ‘12–14’ and ‘more than 14’. The scoring algorithm derives estimates of weekly consumption from the product of frequency of consumption and mean quantity consumed (Supporting information, Table S1).

#### Secondary outcome measures

Secondary outcome measures included alcohol consumption at month 6 and AUDIT‐C score at 6 and 12 months follow‐up; quality of life (EQ‐5D‐5L); service use including use of health and social services, school attendance and contact with criminal justice services at 6 and 12 months; and strengths and difficulties questionnaire scores at 12 months follow‐up.

#### Process outcome measures

We assessed engagement with eBI by remotely monitoring when participants used the app. on their smartphones or accessed the web‐based app. We assessed the fidelity of delivering the PFBA intervention by recording a random sample of 20% of the interventions delivered by each researcher. Two experienced ASBI clinicians applied a behaviour change rating scale (BECCI) to these recordings, as used in previous trials of ASBI, and used the results in supervision with the interventionists to identify strengths and weaknesses.

#### Economic outcome measures

The primary outcome for the economic evaluation was quality‐adjusted life years (QALYs) measured by the EuroQoL questionnaire with five dimensions and five levels (EQ‐5D‐5L). We also collected data on costs of the interventions and the NHS, social care, criminal justice services and other resources used during the 12 months of follow‐up, using a bespoke version of the client service receipt inventory (CSRI) [31].

### Statistical analysis

#### Sample size estimation

To detect a clinically important effect size (Cohen's *d* = 0.3) [32] of PFBA or eBI on alcohol consumption after 12 months with a two‐sided significance level of 5% and statistical power of 80% requires 175 in each of the three groups, and thus a total of 525 analysable participants. Allowing for a 70% follow‐up rate at 12 months we planned to randomize 750 participants. Based on an estimated prevalence of 24.2% of AUDIT‐C scores of at least 3 from an earlier ED survey, and an estimated consent rate of 60%, we planned to approach 5165 potential participants to achieve the target sample of 750. All data requests should be submitted to the corresponding author for consideration. Access to anonymized data may be granted following review. Exclusive use will be retained until the publication of major outputs.

#### Primary analysis

Primary analysis was by treatment allocated using a two‐sided 5% significance level. The primary outcome was alcohol consumption measured by extended AUDIT‐C questionnaire at 12 months post‐randomization. The distribution of this outcome led us to use the cube root transformation to approximate a normal distribution. We then used multivariable regression analysis of covariance, adjusting for baseline alcohol consumption, age, gender and centre (as a random effect), to estimate the differences between groups.

#### Sensitivity analysis

To consider missing primary outcome data, we first analysed only complete cases adjusting for baseline consumption, age, gender and centre. Secondly, we extended this by using ‘last outcome carried forward’ to infer missing data. Thirdly, we used multiple imputation, stratifying the model by allocated group and including demographic, baseline and month 6 outcomes to adjust the primary outcome. We undertook 30 such imputations and averaged the results. We conducted another sensitivity analysis to explore the possibility that data were missing not at random using a pattern mixture approach adjusted for baseline covariates, as proposed by White *et al*. and operationalized by the STATA command ‘rctmiss’ [33, 34].

#### Secondary analysis

Similarly, we used regression, linear or logistic, as appropriate, to model the relationship between observed outcomes and baseline variables, including allocated group. We extended these models to assess the effect of adherence to the interventions on the observed outcomes. We complemented this classical hypothesis testing by estimating the corresponding Bayes factors, which quantify the support for one hypothesis over another by the ratio of the marginal likelihood of two competing hypotheses—the alternative hypothesis that PFBA (or eBI) differs in outcome from screening alone and the alternative hypothesis that it does not differ.

### Cost‐effectiveness analysis

We estimated the incremental cost‐effectiveness of the two interventions relative to screening alone from the perspective of both the NHS and personal social services (PSS) and society in general. Costs of screening and delivering the interventions were estimated by monitoring and valuing the resources used in each arm of the trial and effects on NHS and beyond from the CSRI data; costs are reported from 2014, the beginning of the trial period. The NHS and PSS perspectives included treatment to reduce drinking [e.g. child and adolescent mental health services (CAMHS)], spending time in care (e.g. foster care), being admitted to hospital, using hospital services [e.g. accident and emergency (A&E)] and using community services [e.g. the general practitioner (GP)]. The societal perspective also included educational measures (e.g. exclusions and involvement with the police, e.g. court attendance). We valued these effects from local unit costs, supplemented by national unit costs (Supporting information, Table S2); it should be noted that CSRI data related only to the patients themselves and did not capture data relating to the parent's time and their costs incurred when dealing with their children. Intervention costs were calculated using staff salary costs and time spent training. Additional training costs included preparation, travel, accommodation and parking. These costs were then divided by the number of patients in the eBI and PFBA groups to generate a per patient intervention cost (Supporting information, Table S3). A per patient cost for the app. development and management, iPads and data storage were also included for the eBI patients.

Where EQ‐5D‐5L utility values were missing the mean utility values for that arm and time‐point (baseline, 6 months, 12 months) were imputed. Where resource use was missing it was assumed that no resource had been consumed.

We expressed the cost‐effectiveness as incremental cost per QALY gained and compared that with the willingness to pay (WTP) threshold of £20–30 k recommended by the National Institute of Health and Care Excellence (NICE) [23]. To investigate sampling or joint uncertainty in costs and effects, we applied sensitivity analysis using non‐parametric bootstrapping and presented results via cost‐effectiveness planes.

The bootstrapping methodology randomly resampled 1000 simulated replications from the original cost and effect data from each trial arm creating incremental cost‐effectiveness ratios (ICERs) for each replication. These ICERs were then used to calculate the proportion that were cost‐effective at the WTP threshold of £20 000. The cost‐effectiveness plane plots these resampled incremental cost and effect differences.

## RESULTS

### Recruitment and follow‐up

**FIGURE 1:**
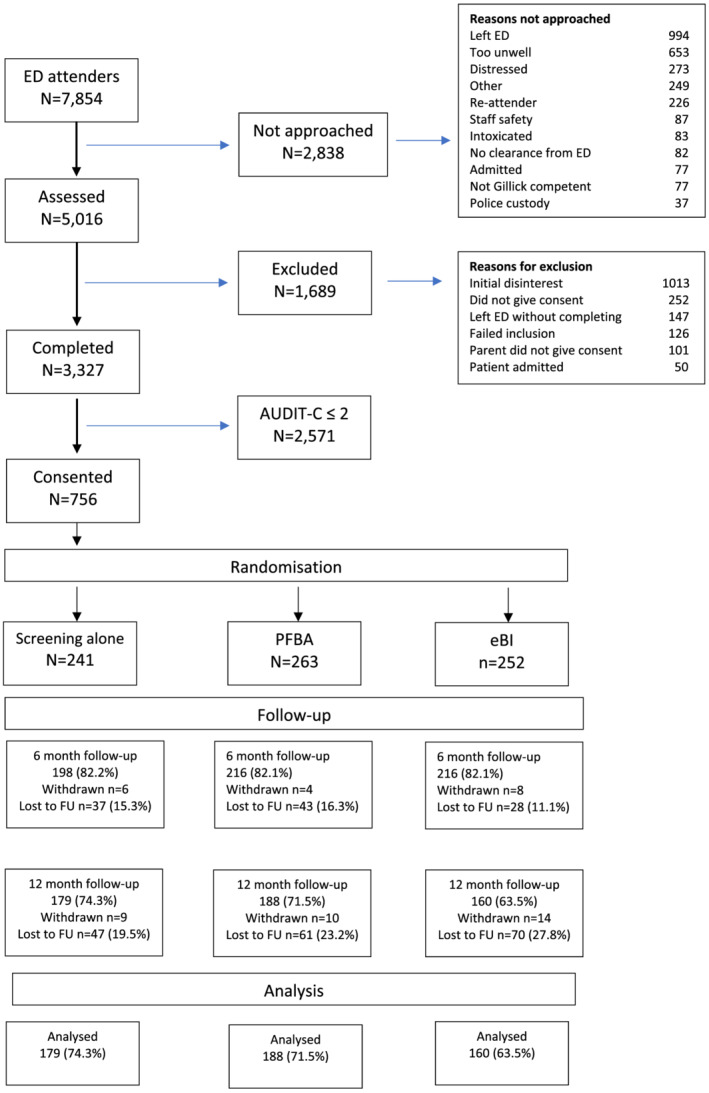
Consort diagram showing actual recruitment and intervention. ED = emergency department; FU = follow‐up; PFBA = personalized feedback and brief advice

Of the 7854 participants in the target age group who attended EDs during the screening period we succeeded in approaching 5016 (63.8%), 3327 (66.4%) of whom consented to be screened to participate in the trial. Of these participants, 756 (22.7%) scored ≥ 3 on AUDIT‐C and consented to take part in the trial (Figure 1). We randomized 263 to PFBA, 252 to eBI and 241 to screening alone. Their mean age was 16.1 years; 50.2% were female and 84.9% were white. Table 2 shows the baseline characteristics of trial participants; as we expected from our rigorous randomization procedure, this shows no real differences between groups. Figure 1 displays the reasons why we could not approach the other 2838. At 6 months, 630 (83%) in total completed assessments; at 12 months, 527 (70%) did so, thus achieving our target of 525.

**TABLE 2:** Demographic and baseline characteristics by allocated group

	Screening alone (SA) (*n* = 241)	PFBA (*n* = 263)	eBI (*n* = 252)
Mean age in years (SD)	16.1 (0.9)	16.0 (0.9)	16.1 (0.9)
Mean age of first drink (SD)	13.4 (2.1)	13.7 (1.7)	13.3 (2.2)
Male *n* (%)	125 (51.9)	127 (48.3)	124 (49.2)
Ethnicity			
White: *n* (%)	207 (85.9)	223 (84.8)	211 (84.1)
Black: *n* (%)	9 (3.7)	14 (5.3)	15 (5.9)
Asian: *n* (%)	3 (1.2)	5 (1.9)	1 (0.3)
Other: *n* (%)	22 (9.2)	21 (8.0)	24 (9.7)
Smoker: *n* (%)	97 (40.3)	95 (36.1)	96 (38.2)
Alcohol use			
Mean weekly alcohol consumption (SD)^a^	5.01 (7.82)	4.33 (8.96)	4.55 (7.43)
Mean AUDIT‐C score (SD)	4.86 (1.80)	4.77 (1.93)	4.87 (1.88)
Heavy alcohol use at least monthly: *n* (%)^b^	91 (37.8)	91 (34.6)	106 (42.1)
Ever intoxicated: *n* (%)^c^	194 (80.7)	211 (80.2)	208 (82.5)
Intoxicated in past 12 months: *n* (%)^c^	170 (70.6)	186 (70.9)	182 (72.4)
Intoxicated in past 30 days: *n* (%)^c^	76 (31.4)	81 (30.7)	69 (27.2)
Alcohol‐related problems			
Ever fighting: *n* (%)	41 (17.1)	46 (17.6)	57 (22.6)
Ever accident or injury: *n* (%)	79 (32.8)	85 (32.4)	84 (33.3)
Ever parent problem: *n* (%)	41 (17.0)	39 (15.0)	47 (18.7)
Ever peer problem: *n* (%)	55 (22.8)	62 (23.4)	71 (28.3)
Ever school problem: *n* (%)	24 (10.0)	47 (17.9)	38 (15.1)
Ever victim of theft: *n* (%)	38 (15.9)	46 (17.6)	44 (17.5)
Ever police problem: *n* (%)	18 (7.5)	31 (11.8)	39 (15.5)
Ever hospitalized: *n* (%)	36 (14.9)	35 (13.3)	31 (12.4)
Ever unprotected sex: *n* (%)	46 (19.1)	39 (14.9)	61 (24.3)
Ever regretted sex: *n* (%)	32 (13.4)	39 (14.8)	47 (18.8)
Strengths and difficulties			
Mean total score (SD)	12.0 (5.62)	11.9 (6.06)	12.6 (5.87)
Mean emotional symptom score (SD)	3.37 (2.52)	3.27 (2.47)	3.37 (2.52)
Mean conduct problem score (SD)	2.28 (1.71)	2.31 (1.66)	2.61 (1.83)
Mean hyperactivity score (SD)	4.24 (2.19)	4.33 (2.30)	4.39 (2.33)
Mean peer problem score (SD)	2.17 (1.68)	2.02 (1.73)	2.28 (1.63)
Mean prosocial behaviour score (SD)	7.29 (1.94)	7.31 (2.01)	7.47 (2.00)

^a^ Measured in standard units of alcohol (equal to 8 g ethanol).

^b^ Defined as 6 or more standard units on a single drinking episode.

^c^ Intoxicated in respondent's judgement. SA = screening alone; PFBA = personalized feedback and brief advice; eBI = electronic brief intervention; SD = standard deviation; AUDIT‐C = Alcohol Use Disorders Identification Test: consumption.

### Clinical outcomes

#### Primary outcome

Alcohol consumption at 12 months were observed to be higher across groups relative to baseline, but there were no significant differences between groups (Tables 3 and 4). As our sensitivity analyses did not deviate from the complete case analysis, that is what we present (Supporting information, Tables S4 and S5).

**TABLE 3:** Adjusted outcome means and 95% confidence intervals at 6 and 12 months by allocated group: complete case analysis

	Screening alone SA (*n* = 179)	PFBA (*n* = 188)	eBI (*n* = 160)
Alcohol use			
Weekly alcohol consumption^a^			
Month 6	2.42 (1.84; 3.11)	2.13 (1.62; 2.74)	2.33 (1.77; 3.00)
Month 12	2.99 (2.38; 3.70)	3.56 (2.90; 4.32)	3.18 (2.50; 3.97)
AUDIT‐C score			
Month 6	4.64 (4.17; 5.11)	4.30 (3.85; 4.75)	4.64 (4.18; 5.11)
Month 12	5.04 (4.65; 5.44)	5.25 (4.87; 5.63)	5.12 (4.70; 5.54)
Strengths and difficulties (12 months only)			
Total score	11.0 (10.2; 11.7)	10.9 (10.2; 11.6)	10.9 (10.1; 11.6)
Emotional symptom score	3.14 (2.82; 3.46)	3.23 (2.91; 3.54)	3.09 (2.75; 3.43)
Conduct problem score	1.90 (1.70; 2.10)	1.74 (1.55; 1.94)	1.86 (1.65; 2.07)
Hyperactivity score	3.54 (3.23; 3.84)	3.73 (3.43; 4.02)	3.87 (3.55; 4.19)
Peer problem score	2.30 (2.06; 2.54)	2.21 (1.97; 2.44)	2.05 (1.80; 2.30)
Prosocial behaviour score	7.91 (7.66; 8.16)	8.21 (7.97; 8.45)	7.75 (7.49; 8.01)

^a^ Measured in standard units of alcohol (equal to 8 g ethanol). PFBA = personalized feedback and brief advice; eBI = electronic brief intervention; AUDIT‐C = Alcohol Use Disorders Identification Test: consumption.

**TABLE 4:** Adjusted mean outcome differences from screening alone and 95% CIs by allocated group

Alcohol use	PFBA	eBI
Weekly alcohol consumption^a^		
Month 6	−0.286 (−0.903; 0.478)	−0.0886 (−0.756; 0.737)
Month 12	0.570 (−0.362; 1.70)	0.186 (−0.714; 1.30)
AUDIT‐C score		
Month 6	−0.334 (−0.858; 0.189)	0.00685 (−0.528; 0.542)
Month 12	0.206 (−0.334; 0.747)	0.0818 (−0.488; 0.652)
Strengths and difficulties at 12 months		
Total score	−0.0170 (−1.02; 0.981)	−0.0998 (−1.14; 0.945)
Emotional symptom score	0.0891 (−0.340; 0.518)	−0.0523 (−0.501; 0.396)
Conduct problem score	−0.161 (−0.436; 0.113)	−0.0426 (−0.330; 0.245)
Hyperactivity score	0.193 (−0.232; 0.618)	0.334 (−0.111; 0.779)
Peer problem score	−0.0901 (−0.386; 0.206)	−0.249 (−0.559; 0.0608)
Prosocial behaviour score	0.293 (−0.0406; 0.626)	−0.165 (−0.514; 0.183)

^a^ Measured in standard units of alcohol (equal to 8 g ethanol. PFBA = personalized feedback and brief advice; eBI = electronic brief intervention; AUDIT‐C = Alcohol Use Disorders Identification Test: consumption.

#### Secondary outcomes

There were no significant differences between groups on any secondary outcome, notably scores for AUDIT‐C and strengths and difficulties questionnaire (Table 4).

We estimated the Bayes factor comparing PFBA with screening alone as 0.08 [standard error (SE) = 0.36], and that comparing eBI with screening alone as 0.08 (SE = 0.16). These results suggest that the reported effects are due to a lack of effect rather than a lack of evidence of an effect.

Exploratory analysis of potential predictors of alcohol consumption after 12 months identified several significant predictors: higher baseline alcohol consumption, lower age of first drink, older age at recruitment, male gender, greater alcohol expectancy and more alcohol‐related problems (Supporting information, Table S6). Of those allocated to eBI, 84 (33%) engaged with the intervention at least once after leaving the ED, for a median of 126 secs (interquartile range from 0 to 822), but we found no association between this engagement and alcohol consumption at 12 months.

### Cost‐effectiveness

Table 5 compares both PFBA and eBI with screening alone from the perspective of NHS and PSS; Table 6 does so from the societal perspective. eBI was dominated by screening alone from both perspectives, in the sense that it cost more and had a very slightly lesser effect on AUDIT‐C; but neither effect was significant.

**TABLE 5:** Results of cost‐effectiveness analysis from perspective of NHS and PSS

	Screening alone (SA)	eBI	Difference
	Mean (bootstrapped standard deviation)		eBI–SA
Total costs	£1552 (£6019)	£1953 (£6960)	£401 (−£1424, +£2346)
Total QALYS	0.900 (0.096)	0.892 (0.105)	−0.008 (−0.037, o + 0.019)
ICER (£/QALY gained)	Screening dominates eBI		
	Screening alone (SA)	PFBA	Difference PFBA–SA
Total costs	£1553 (£6019)	£1571 (£6114)	£18 (−£1752, +£1586)
Total QALYS	0.900 (0.096)	0.903 (0.089)	0.003 (−0.023, +0.026)
ICER (£/QALY gained)	£6213 (−£736 843, +£812 884)		

^a^ Measured in standard units of alcohol (equal to 8 g ethanol). PFBA = personalized feedback and brief advice; eBI = electronic brief intervention; QALYs = quality‐adjusted life years; ICER = incremental cost‐effectiveness ratio; NHS = National Health Service; PSS = personal social services.

**TABLE 6:** Results of cost‐effectiveness analysis from societal perspective

	Screening alone (SA)	eBI	Difference
Total costs	£1703 (£6049)	£2110 (£7040)	£406 (−£1334, £2331)
Total QALYS	0.900 (SD 0.096)	0.892 (SD 0.105)	−0.008 (−0.038, 0.021)
ICER (£/QALY gained)	Screening dominates eBI		
	Screening alone (SA)	PFBA	Difference
Total costs	£1703 (£6049)	£1726 (£6152)	£22 (−£1860, £1663)
Total QALYS	0.900 (0.096)	0.903 (0.089)	0.003 (−0.023, 0.028)
ICER (£/QALY gained)	£7580 (−£1 088 865, +£794 373)		

PFBA = personalized feedback and brief advice; eBI = electronic brief intervention; QALYs = quality‐adjusted life years; ICER = incremental cost‐effectiveness ratio.

**FIGURE 2:**
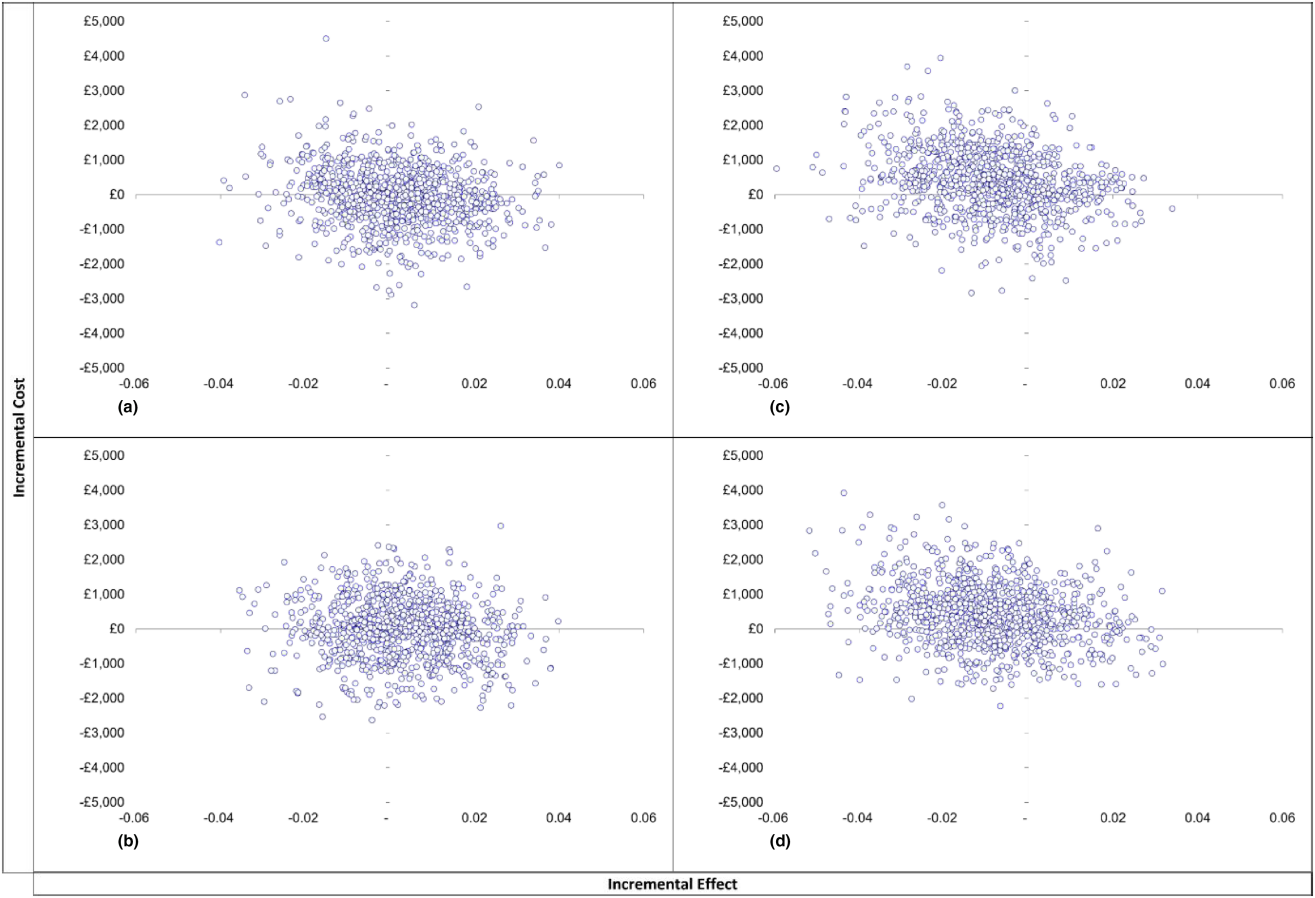
Cost‐effectiveness plane comparing personalized feedback and brief advice (PFBA) with screening alone (SA) from National Health Service (NHS) + personal social services (PSS) (a) and societal (b) perspectives, and electronic brief intervention (eBI) with SA from NHS + PSS (c) and societal (d) perspectives

**FIGURE 3:**
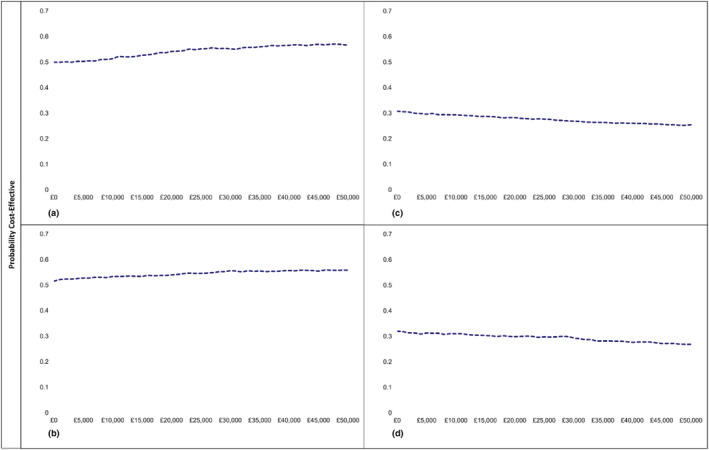
Cost‐effectiveness acceptability curve comparing personalized feedback and brief advice (PFBA) with screening alone (SA) from National Health Service (NHS) + personal social services (PSS) (a) and societal (b) perspectives and electronic brief intervention (eBI) with SA from NHS + PSS (c) and societal (d) perspectives

PFBA yielded incremental cost‐effectiveness ratios (ICERs) of £6213 (−£736 843 to £812 884) per QALY for NHS and PSS and £7580 (−£1 088 865 to £794 373) for society. At first sight, these ICERs are markedly less than the ‘willingness to pay’ threshold of £20 000 generally used by NICE. However, these apparently encouraging ICERs result from dividing very small differences in costs by very small differences in QALYs. As we have already seen, the corresponding Bayes factors do not approach statistical significance. Similarly, Figure 2a–d shows that the cost‐effectiveness plane (CEP) showing the distribution of both incremental NHS and PSS and societal costs and effects of PFBA and eBI has wide variability. Thus, the related cost‐effectiveness acceptability curve (CEAC) of PFBA from the NHS + PSS perspective (Figure 3a) shows that only 54% of re‐samples of PFBA versus screening alone were cost‐effective at the £20 000 threshold. The corresponding CEAC from the societal perspective (Figure 3b) also estimated that only 54% of re‐samples were cost‐effective.

The CEPs for eBI from both perspectives also displayed wide variability. The CEAC for eBI from the perspective of NHS and PSS (Figure 3c) estimated the chance of cost‐effectiveness as only 30%, while that from the societal perspective (Figure 3d) estimated that chance as only 28%.

## DISCUSSION

### Summary of findings

As we expected, all three groups were well matched by our randomization procedure. To minimize the risk of bias, our analysis controlled for baseline covariates known to influence outcome. We then found no significant differences in either primary or secondary outcomes. *Post‐hoc* Bayesian analysis supported the null hypothesis that PFBA and eBI are as effective as screening alone in reducing alcohol consumption in high‐risk adolescent drinkers. Economic analysis also supported the null hypothesis that PFBA and eBI are not cost‐effective compared to screening alone in this population. We observed little difference in resource use between groups, despite a few large social care costs.

### Interpretation

These findings are similar to those of our linked trial targeting abstinent or low‐risk adolescent drinkers in the same ED settings which we have reported elsewhere [15]. However, the absence of benefit of conventional ASBI over screening alone contrasts with earlier published ED trials in this age group [11, 12, 13] and adults [9, 10]. It is notable that most previous trials in adolescents were conducted in single sites, whereas this trial (SIPS Junior) was conducted in 10 typical EDs across England. Previous early positive findings in ASBI efficacy trials have not generally translated into equivalent findings in larger and more pragmatic trials. It may be that ASBI interventions are less well implemented in pragmatic trials such as SIPS Junior. However, we made extensive efforts to standardize the delivery of interventions and assess fidelity. Therefore, the lack of effectiveness of ASBI has important implications for practice, as pragmatic trials try to implement innovations in the real world rather than in the ‘ideal’ laboratory environment.

The complete absence of benefit of eBI also contrasts with previously published research on eBI in young people and adults, where our earlier work suggested evidence of efficacy [14]. There are several possible explanations for this difference in findings. None of the previous eBI trials in our systematic review included smartphone‐delivered eBI; most were internet‐delivered. It may be that participants engage differently with smartphone alcohol apps than with the internet. Recently published smartphone‐delivered eBI trials showed no impact on drinking, and support our finding [35, 36].

We found that it is possible to implement ASBI in EDs and engage most of the target population in alcohol screening and identification of hazardous and harmful alcohol use. We also identified a large proportion of adolescent ED attenders who were drinking at hazardous or harmful levels. Therefore, ED remains a useful setting in which to identify adolescent risk drinkers when appropriate staff and methods are available to do so. However, we have previously demonstrated that ASBI is difficult to implement in the typical ED without additional trained alcohol staff [37].

### Strengths and limitations

We exceeded our target recruitment and the planned follow‐up rate at 6 months, and we achieved the planned follow‐up rate at the primary outcome time‐point (12 months). This meant that the trial was adequately powered to detect clinically meaningful differences in alcohol consumption at the primary outcome point of 12 months. We also exceeded our expected eligibility and consent conversion rates, and thus recruited a representative sample of patients in the target age range.

There is a question of whether our use of AUDIT‐C rather than TLFB to derive consumption may have masked small but important changes over time. The decision was pragmatic and at the time of protocol development there was no evidence that a self‐administered TLFB was reliable and valid in an adolescent population. In addition, we conducted an analysis to explore levels of agreement between consumption derived from AUDIT‐C and TLFB in advance of undertaking the study and found acceptable levels of agreement. Taken together with evidence from Bradley *et al*. [30], that AUDIT‐C is sensitive to change over time, makes us confident that the use of AUDIT‐C has not masked small but important changes.

However, only a third of eBI participants engaged with the eBI app. after leaving the ED. Poor app. engagement is a common issue for health apps, the vast majority of which are not used a month after being downloaded [38]. Although numerous strategies already exist to promote engagement [39], further research is needed to identify app. features and other factors that promote engagement and the extent to which they promote behaviour change.

From a cost perspective, the implementation costs of the interventions were spread across each of the participants in the respective arms; in the real world, these costs would become less per person as more people utilized the intervention. Given the relatively small implementation costs of the intervention, it is unlikely that this would affect the conclusions of this study.

## CONCLUSIONS

ASBI and eBI are not effective or cost‐effective compared to screening alone in reducing alcohol consumption in high‐risk drinking adolescents. Hence, this trial does not support the implementation of these interventions. Our previous pragmatic trials in EDs in adults found that more intensive alcohol interventions are no better than simple alcohol screening and feedback [37]. However, previous research with young people has shown that alcohol screening can reduce drinking [40]. Screening alone potentially raises awareness of hazardous drinking and may be sufficient to initiate behaviour change [41]. Screening is also able to identify patients drinking at harmful and dependent levels who may benefit from referral to more specialist services or require safeguarding procedures. Based on current evidence, therefore, alcohol screening and simple feedback may be the best available intervention for high‐risk drinking adolescents presenting to ED. For adolescents with alcohol dependence or complex needs or where there are significant safeguarding concerns, current clinical guidelines advocate referral to specialist alcohol and/or mental health services [42], which were not tested in this trial, but seems appropriate based on a precautionary principle.

## DECLARATION OF INTERESTS

J.S. reports competing interests with Molteni Farma, and grants from Mundipharma, Camurus and Accord Pharma outside the submitted work. K.E.K., N.‐B.D., P.R., P.‐H.T., P.C. and R.I.T. report grants from NIHR during the conduct of the study. S.B. works at the Institute of Alcohol Studies, which receives funding from the Alliance House Foundation. K.E.K. is now a panel member of NIHR PGfAR, which funded this study, but not at the time the work was conducted. P.C. received additional funding from the NIHR Yorkshire and Humber Clinical Research Network to support the development and delivery of clinical and applied health research across the region. The other authors have no conflicts of interest.

## AUTHOR CONTRIBUTIONS

**Paolo Deluca:** Conceptualization; funding acquisition; methodology; project administration; software. **Simon Coulton:** Conceptualization; data curation; formal analysis; funding acquisition; investigation; methodology; validation. **M Fasihul Alam:** Formal analysis. **Sadie Boniface:** Investigation; project administration; supervision. **Kim Donoghue:** Project administration; supervision. **Eilish Gilvarry:** Conceptualization; funding acquisition; methodology; supervision. **Eileen Kaner:** Conceptualization; funding acquisition; methodology; project administration; supervision. **Ellen Lynch:** Project administration; supervision. **Ian Maconochie:** Conceptualization; funding acquisition; methodology. **Paul McArdle:** Conceptualization; funding acquisition; methodology. **Ruth McGovern:** Methodology; project administration; resources; supervision. **Dorothy Newbury‐Birch:** Conceptualization; funding acquisition; methodology; project administration; supervision. **Robert Patton:** Conceptualization; project administration; software; supervision. **Tracy Pellatt‐Higgins:** Formal analysis; investigation; methodology; visualization. **Ceri Phillips:** Formal analysis; methodology; resources. **Thomas Phillips:** Conceptualization; funding acquisition; methodology; project administration; resources; software; supervision. **Rhys Pockett:** Formal analysis; methodology. **Ian Russell:** Conceptualization; funding acquisition; methodology; project administration; validation. **John Strang:** Conceptualization; funding acquisition; project administration. **Colin Drummond:** Conceptualization; funding acquisition; methodology; project administration; supervision.

## CLINICAL TRIAL REGISTRATION

International standard randomized controlled trial number (ISRCTN) 45 300 218.

## Supporting information


**Table S1:** Derived alcohol consumption from the extended AUDIT‐C Multiply Q1 factor by Q2 factor weekly consumption
**Table S2:** Health economics resource unit costs (costs reported in £ 2014)
**Table S3:** Intervention costs
**Table S4:** Demographic and outcome variables by whether followed up at month 12
**Table S5:** Sensitivity analysis for missing primary outcomes, weekly alcohol consumed at month 12.
**Table S6:** Exploratory linear regression of mean weekly alcohol consumption at month 12 (transformed before analysis using cube root) on pre‐randomization factors
