# Supplementary material for: Effectiveness and cost‐effectiveness of face‐to‐face and electronic brief interventions versus screening alone to reduce alcohol consumption among high‐risk adolescents presenting to emergency departments: three‐arm pragmatic randomized trial (SIPS Junior high risk trial)
Source: Addiction. 2022 Apr 12;117(8):2200–14. doi: 10.1111/add.15884 (PMC9540754; doi:10.1111/add.15884)
Supplement: Supplementary file 1 — Table S1: Derived alcohol consumption from the extended AUDIT‐C Multiply Q1 factor by Q2 factor weekly consumption Table S2: Health economics resource unit costs (costs reported in £ 2014) Table S3: Intervention costs Table S4: Demographic and outcome variables by whether followed up at month 12 Table S5: Sensitivity analysis for missing primary outcomes, weekly alcohol consumed at month 12. Table S6: Exploratory linear regression of mean weekly alcohol consumption at month 12 (transformed before analysis using cube root) on pre‐randomization factors [file ADD-117-2200-s001.docx]

**Supplementary Table 1: Derived alcohol consumption from the extended AUDIT-C
Multiply Q1 factor by Q2 factor weekly consumption**

|  | **Factor** |
| --- | --- |
| Q1. Over the past **6 months**, how often have you had a drink containing alcohol?  Never Monthly or less 2 to 4 times a month 2 to 3 times a week 4 to 5 times a week 6 or more times a week  Q2. How many standard drinks containing alcohol do you drink on a typical day you are drinking?  1 to 2 3 to 4 5 to 6 7 to 9 10 to 11 12 to 14 More than 14 | 0  0.25  3  10  18  25  1.5  3.5  5.5  8  10.5  13  16 |

**Supplementary Table 2: Health economics resource unit costs (costs reported in £ 2014)**

| **Contact with Health Professionals** | **Unit Cost (£)** | **Details of cost components** | **Reference** |
| --- | --- | --- | --- |
| Consultation with GP | £46.00 | 11.7min consultation, including direct care staff costs, with qualification costs. (Curtis, 2014; pp.195) | Curtis, L. (2014) Unit Costs of Health and Social Care 2014, Personal Social Services Research Unit, University of Kent, Canterbury. |
| Seen the Practice Nurse | £13.69 | £53 per hour for face-to-face consultation, including qualification costs. Average consultations last 15.5minutes. Cost calculated as follows - £53/60 x 15.5 (Curtis, 2014; pp.192) | Curtis, L. (2014) Unit Costs of Health and Social Care 2014, Personal Social Services Research Unit, University of Kent, Canterbury. |
| Seen the Health Visitor | £19.69 | £76 per hour of patient related work, including qualification costs. Average consultation time for a PRACTICE NURSE (15.5mins). So, (£1.27 per minute x 15.5 = £19.69) (Curtis, 2014; pp. 187, 189, 192) | Curtis, L. (2014) Unit Costs of Health and Social Care 2014, Personal Social Services Research Unit, University of Kent, Canterbury. |
| Hospital Inpatient Elective Stay | £3,375.42 | National average unit cost | Department of Health (2014) Reference Costs available from <https://www.gov.uk/government/uploads/system/uploads/attachment_data/file/380322/01_Final_2013-14_Reference_Costs_publication_v2.pdf> |
| Hospital Inpatient Elective Stay - excess bed days | £326.90 | National average unit cost | Department of Health (2014) Reference Costs available from <https://www.gov.uk/government/uploads/system/uploads/attachment_data/file/380322/01_Final_2013-14_Reference_Costs_publication_v2.pdf> |
| Hospital Inpatient Non-Elective SHORT Stay (1-3 days) | £602.52 | National average unit cost | Department of Health (2014) Reference Costs available from <https://www.gov.uk/government/uploads/system/uploads/attachment_data/file/380322/01_Final_2013-14_Reference_Costs_publication_v2.pdf> |
| Hospital Inpatient Non-Elective Long Stay (4 - 6 days) | £2,837.31 | National average unit cost | Department of Health (2014) Reference Costs available from <https://www.gov.uk/government/uploads/system/uploads/attachment_data/file/380322/01_Final_2013-14_Reference_Costs_publication_v2.pdf> |
| Hospital Inpatient Non-Elective - excess bed days (incurred for stays greater than 6 days) | £275.05 | National average unit cost | Department of Health (2014) Reference Costs available from <https://www.gov.uk/government/uploads/system/uploads/attachment_data/file/380322/01_Final_2013-14_Reference_Costs_publication_v2.pdf> |
| Hospital Day case | £697.55 | National average unit cost | Department of Health (2014) Reference Costs available from <https://www.gov.uk/government/uploads/system/uploads/attachment_data/file/380322/01_Final_2013-14_Reference_Costs_publication_v2.pdf> |
| Regular day/night admission | £400.23 | National average unit cost | Department of Health (2014) Reference Costs available from <https://www.gov.uk/government/uploads/system/uploads/attachment_data/file/380322/01_Final_2013-14_Reference_Costs_publication_v2.pdf> |
| Accident & Emergency visit | £123.67 | National average unit cost | Department of Health (2014) Reference Costs available from <https://www.gov.uk/government/uploads/system/uploads/attachment_data/file/380322/01_Final_2013-14_Reference_Costs_publication_v2.pdf> |
| Outpatient department visit | £109.00 | Weighted average of all outpatient attendences. (Curtis, 2014; pp. 111) | Curtis, L. (2014) Unit Costs of Health and Social Care 2014, Personal Social Services Research Unit, University of Kent, Canterbury. |
| Consulted or visited other health care professional | £34.67 | Calculated average of hospital-based health care staff, including qualification costs - Physio (£37), OT (£36), SALT (£37), Dietitian (£37), Radiographer (£38), Allied health professional support worker (£23) = £34.67. (Curtis, 2014; pp. 235-241) ASSUMED HOUR CONSULTATION - given that there's no average consultation time | Curtis, L. (2014) Unit Costs of Health and Social Care 2014, Personal Social Services Research Unit, University of Kent, Canterbury. |
| Community Services | Unit Cost (£) | Details of cost components | Reference |
| Visited Optician | £21.00 | Average "Specsavers" cost | Curtis, L. (2014) Unit Costs of Health and Social Care 2014, Personal Social Services Research Unit, University of Kent, Canterbury. |
| Visited Family Therapist | £45.83 | £50 per hour. Average consultations last 55 minutes. Cost calculated as follows - (£50/60) x 55 = £45.83 (Curtis, 2014; pp.51) | Curtis, L. (2014) Unit Costs of Health and Social Care 2014, Personal Social Services Research Unit, University of Kent, Canterbury. |
| Visited Individual Therapist | £45.83 | £50 per hour. Average consultations last 55 minutes. Cost calculated as follows - (£50/60) x 55 = £45.83 (Curtis, 2014; pp.51) | Curtis, L. (2014) Unit Costs of Health and Social Care 2014, Personal Social Services Research Unit, University of Kent, Canterbury. |
| Visited Psychiatrist/Psychologist | £69.00 | £138 per hour for client related work, including qualification costs. Average consultations last 30 minutes (assumption). Cost calculated as follows - (£138/60) x 30 = £69.00 (Curtis, 2014; pp.183) | Curtis, L. (2014) Unit Costs of Health and Social Care 2014, Personal Social Services Research Unit, University of Kent, Canterbury. |
| Visited Social Worker | £39.50 | £79 per hour for client related work, including qualification costs. Average consultations last 30 minutes (assumption). Cost calculated as follows - (£79/60) x 30 = £39.50 (Curtis, 2014; pp.207) | Curtis, L. (2014) Unit Costs of Health and Social Care 2014, Personal Social Services Research Unit, University of Kent, Canterbury. |
| Home visit optician | £31.00 | Estimated cost of optician visit at £21.00 + £10.00 travel costs |  |
| Home visit family therapist | £70.83 | £50 per hour. Average consultations last 55 minutes + 15 minutes each way travel costs (assumption). Cost calculated as follows - (£50/60) x 85 = £70.83 (Curtis, 2014; pp.51) | Curtis, L. (2014) Unit Costs of Health and Social Care 2014, Personal Social Services Research Unit, University of Kent, Canterbury. |
| Home visit individual therapist | £70.83 | £50 per hour. Average consultations last 55 minutes + 15 minutes each way travel costs (assumption). Cost calculated as follows - (£50/60) x 85 = £70.83 (Curtis, 2014; pp.51) | Curtis, L. (2014) Unit Costs of Health and Social Care 2014, Personal Social Services Research Unit, University of Kent, Canterbury. |
| Home visit Psychiatrist/Psychologist | £138.00 | £138 per hour for client related work, including qualification costs. Average consultations last 30 minutes + 15 minute each way travel (assumption). Cost calculated as follows - (£138/60) x 60 = £138.00 (Curtis, 2014; pp.183) | Curtis, L. (2014) Unit Costs of Health and Social Care 2014, Personal Social Services Research Unit, University of Kent, Canterbury. |
| Home visit Social Worker | £39.50 | £79 per hour for client related work, including qualification costs. Average consultations last 30 minutes (assumption). Cost calculated as follows - (£79/60) x 30 = £39.50 (Curtis, 2014; pp.207) | Curtis, L. (2014) Unit Costs of Health and Social Care 2014, Personal Social Services Research Unit, University of Kent, Canterbury. |
| Home visit other |  |  |  |
| Home visits: | Unit Cost (£) | Details of cost components | Reference |
| GP | £91.26 | 11.4 min home visit, plus 12 minutes travel time per visit on average. 1 minute of GP time is costed at £3.90 (including direct care saff costs with qualification costs pg. 195), this is multiplied by 23.4 to estimate cost of home visit. (Curtis, 2014; pp. 194-195) | Curtis, L. (2014) Unit Costs of Health and Social Care 2014, Personal Social Services Research Unit, University of Kent, Canterbury. |
| Community Nurse | £30.25 | £66 per hour of patient related work, including qualification costs. Average consultation time for a PRACTICE NURSE (15.5mins), and average travel time for a GP (12mins) used to calculate cost per visit. So, (£1.10 per minute x 15.5 = £17.05) + (£1.10 x 12 = £13.20) = £30.25 (Curtis, 2014; pp. 187, 192, 194-195) | Curtis, L. (2014) Unit Costs of Health and Social Care 2014, Personal Social Services Research Unit, University of Kent, Canterbury. |
| Practice Nurse | £24.29 | £53 per hour for face-to-face consultation at GP surgery, including qualification costs. Average consultations last 15.5minutes - assumed to be the same for home visits. Average travel time for a GP (12mins) used to calculate travel costs. So, (53/60 x 15.5 = £13.69) + (53/60 x 12 = £10.60) = £24.29 (Curtis, 2014; pp. 192, 194-195) | Curtis, L. (2014) Unit Costs of Health and Social Care 2014, Personal Social Services Research Unit, University of Kent, Canterbury. |
| Health Visitor | £34.93 | £76 per hour of patient related work, including qualification costs. Average consultation time for a PRACTICE NURSE (15.5mins), and average travel time for a GP (12mins) used to calculate cost per visit. So, (£1.27 per minute x 15.5 = £19.69) + (£1.27 x 12 = £15.24) = £34.93 (Curtis, 2014; pp. 187, 189, 192, 194-195) | Curtis, L. (2014) Unit Costs of Health and Social Care 2014, Personal Social Services Research Unit, University of Kent, Canterbury. |
| Other health care professional | £48.48 | Calculated average of community-based health care staff, including qualification costs - Physio (£36), OT (£36), SALT (£36), Palliative Care Nurse specialist (£74), clinical support worker (£20) = £40.40. Then added travel costs - estimated by using GP travel time of 12 mins. So, additional travel cost = (40.40/60 x 12 = £8.08) (Curtis, 2014; pp. 235-241) ASSUMED HOUR CONSULTATION - given that there's no average consultation time. | Curtis, L. (2014) Unit Costs of Health and Social Care 2014, Personal Social Services Research Unit, University of Kent, Canterbury. |
| Drug and Alcohol Services | Unit Cost (£) | Details of cost components | Reference |
| CAMHS F2F | £84.00 | CAMHS cost per hour ranges between £84 and £115 per hour of F2F contact depending on case-mix. Average F2F meeting 60 mins (Assumption). (Curtis, 2014; pp 222-225) | Curtis, L. (2014) Unit Costs of Health and Social Care 2014, Personal Social Services Research Unit, University of Kent, Canterbury. |
| CAMHS Telephone | £16.38 | 11.7min consultation (Average GP telephone consult time) as a proportion of CAMHS cost | Curtis, L. (2014) Unit Costs of Health and Social Care 2014, Personal Social Services Research Unit, University of Kent, Canterbury. |
| Other F2F | £84.00 | Assumed same as CAMHS | Curtis, L. (2014) Unit Costs of Health and Social Care 2014, Personal Social Services Research Unit, University of Kent, Canterbury. |
| Other Telephone | £16.38 | Assumed same as CAMHS | Curtis, L. (2014) Unit Costs of Health and Social Care 2014, Personal Social Services Research Unit, University of Kent, Canterbury. |
| Sick/Truancy Days | Unit Cost (£) | Details of cost components | Reference |
| School exclusion (permanent) | £4,000.00 | https://www.gov.uk/government/uploads/system/uploads/attachment_data/file/269681/Exclusion_from_maintained_schools__academies_and_pupil_referral_units.pdf | <https://www.gov.uk/government/uploads/system/uploads/attachment_data/file/269681/Exclusion_from_maintained_schools__academies_and_pupil_referral_units.pdf> |
| Educational Help | Unit Cost (£) | Details of cost components | Reference |
| Individual tuition at home | £35.00 | Fees average between £29 and £41 per hour | <https://www.journalism.co.uk/press-releases/private-tuition-fees-new-data-on-uk-tutor-rates/s66/a604769> |
| Individual tuition in some classes | £14.26 | £14.26 per hour, based on an average teaching salary of £27,813.5 and 37.5 hours per week of working time. https://getintoteaching.education.gov.uk/funding-and-salary/teacher-salaries | <https://getintoteaching.education.gov.uk/funding-and-salary/teacher-salaries> |
| Lessons in a special unit in school | £14.26 | Assumed same as individual tuition in some classes | <https://getintoteaching.education.gov.uk/funding-and-salary/teacher-salaries> |
| School Professionals | Unit Cost (£) | Details of cost components | Reference |
| School Nurse (per contact) | £53.00 | £53 per contact School-based children's health core (other) services. (Curtis, 2014; pp. 85) | Curtis, L. (2014) Unit Costs of Health and Social Care 2014, Personal Social Services Research Unit, University of Kent, Canterbury. |
| Educational Pyschologist (per contact) | £41.00 | £41 per contact Educational pyschologist. (Curtis, 2014; pp. 156) | Curtis, L. (2014) Unit Costs of Health and Social Care 2014, Personal Social Services Research Unit, University of Kent, Canterbury. |
| Educational Welfare Officer (per contact) | £22.50 | £22.50 per contact Educational welfare officer. Checklist completed by EWO £18 + TAC meetinf attended by EWO £27. Average calculated(Curtis, 2014; pp. 155) | Curtis, L. (2014) Unit Costs of Health and Social Care 2014, Personal Social Services Research Unit, University of Kent, Canterbury. |
| School Counsellor / Health Advisor (per contact) | £41.00 | Assumed same as Educational Pyschologist | Curtis, L. (2014) Unit Costs of Health and Social Care 2014, Personal Social Services Research Unit, University of Kent, Canterbury. |
| Additional Meetings with Tutors (per minute) | £0.24 | £0.2377222222 per minute, based on an average teaching salary of £27,813.5 and 37.5 hours per week of working time. https://getintoteaching.education.gov.uk/funding-and-salary/teacher-salaries | <https://getintoteaching.education.gov.uk/funding-and-salary/teacher-salaries> |
| Other Care | Unit Cost (£) | Details of cost components | Reference |
| Foster Care (days) | £427.86 | £2995 establishment costs per week / 7days = £427.86 per day. (Curtis, 2014; pp 86) | Curtis, L. (2014) Unit Costs of Health and Social Care 2014, Personal Social Services Research Unit, University of Kent, Canterbury. |
| Residential Care (days) | £90.43 | £633 establishment costs per week / 7days = £90.43 per day. (Curtis, 2014; pp 64) | Curtis, L. (2014) Unit Costs of Health and Social Care 2014, Personal Social Services Research Unit, University of Kent, Canterbury. |
| Supported Accomodation (days) | £90.43 | Assumed same as residential care. £633 establishment costs per week / 7days = £90.43 per day. (Curtis, 2014; pp 64) | Curtis, L. (2014) Unit Costs of Health and Social Care 2014, Personal Social Services Research Unit, University of Kent, Canterbury. |
| Other (days) | £90.43 | Assumed same as residential care. £633 establishment costs per week / 7days = £90.43 per day. (Curtis, 2014; pp 64) | Curtis, L. (2014) Unit Costs of Health and Social Care 2014, Personal Social Services Research Unit, University of Kent, Canterbury. |
| Policing and Crime | Unit Cost (£) | Details of cost components | Reference |
| Police contact (spoken to by) | £21.85 | Cost of police constable, per hour "National Policing Guidelines on Charging for Police Services: Mutual Aid Cost Recovery", 2015 pg 16 | NPCC (2015) National Policing Guidelines on Charging for Police Services: Mutual Aid Cost Recovery  available from <http://www.npcc.police.uk/documents/finance/2015/NPCC%20Guidelines%20on%20Charging%20for%20Police%20Services%20Mutual%20Aid.pdf> |
| Court Appearance | £100.00 | Costs vary massively depending on type of court and whether the defendant pleads guilty or goes to trial. We have calculated the cost based on the lowest costed court attendance at a magistrates court. Costs exclude lawyers. | CPS Application for costs against convicted defendants – Scales of Cost  Available from <http://www.cps.gov.uk/legal/a_to_c/costs/annex_1_-_scales_of_cost/> |
| Custody (day) | £418.00 | http://www.mirror.co.uk/news/uk-news/night-prison-cell-costs-more-5555031 | <http://www.mirror.co.uk/news/uk-news/night-prison-cell-costs-more-5555031> |

**Supplementary Table 3: Intervention costs**

| **Item** |  | **No. of patients (eBI and PFBA)** | **Per Patient Cost** |
| --- | --- | --- | --- |
| Training Cost - North | £1,968.82 |  |  |
| Training Prep – North | £122.73 |  |  |
| Training Travel – North | £346.83 |  |  |
| Accomodation – North | £150.00 |  |  |
| Parking - North | £25.40 |  |  |
| Total - North | £2,613.78 | 648 | £4.03 |
| Total - South | £2,638.96 | 446 | £5.92 |
| IPads – eBI | £4,000 |  |  |
| Data handling/storage - eBI | £800 |  |  |
| App development/management -eBI | £32,400 |  |  |
| Total - eBI | £37,200 | 546 | £68.13 |

**Supplementary Table 4: Demographic and outcome variables by whether followed up at month 12**

|  | **Followed Up**  **(n=536)** | **Loss to follow-up (n=220)** |
| --- | --- | --- |
| Allocated group n (%)  eBI  PFBA  SA  Mean age in years (SD)  Male n (%)  White n (%)  Mean weekly alcohol consumption (SD) ^a^  Baseline  Month 6  Mean AUDIT-C score (SD)  Baseline  Month 6  **Strengths and difficulties**  Baseline mean (SD) total score  Baseline mean (SD) emotional symptom score  Baseline mean (SD) conduct problem score  Baseline mean (SD) hyperactivity score  Baseline mean (SD) peer problem score  Baseline mean (SD) prosocial behaviour score | 159 (29.7)  196 (36.6)  181 (33.7)  16.1 (0.92)  253 (47.2)  458 (87.1)  4.19 (6.26)  4.75 (7.17)  4.75 (1.77)  4.59 (2.89)  12.1 (5.91)  3.41 (2.45)  2.28 (1.71)  4.26 (2.30)  2.13 (1.65)  7.55 (1.88) | 93 (42.3)  67 (30.4)  60 (27.3)  16.1 (0.94)  123 (55.9)  184 (85.2)  5.67 (11.4)  4.56 (6.61)  5.03 (1.97)  4.57 (2.89)  12.5 (5.74)  3.16 (2.52)  2.68 (1.78)  4.48 (2.20)  2.21 (1.75)  6.88 (2.16) |

**Supplementary Table 5**: Sensitivity analysis for missing primary outcomes, weekly alcohol consumed at month 12.

|  | **SA** | **PFBA** | **eBI** |
| --- | --- | --- | --- |
| **Mean (95% CI)**  Complete case analysis (n= 536)  Multiple Imputation ^a^ (n=756)  Last Outcome Carried Forward (n=756) | 2.99 (2.38; 3.70)  3.18 (2.55; 3.91)  2.84 (2.36; 3.38) | 3.56 (2.90; 4.32)  3.34 (2.67; 4.12)  3.28 (2.66; 3.98) | 3.18 (2.50; 3.97)  3.08 (2.38; 3.91)  3.03 (2.46; 3.68) |

^a^ Multiple imputations adjusted by baseline demographics, baseline and month 6 outcomes

**Supplementary Table 6: Exploratory linear regression of mean weekly alcohol consumption at month 12 (transformed before analysis using cube root) on pre-randomisation factors**

| **Effect** | **Coefficient (intercept)** | **Estimate** | **F statistic** | **Significance level** |
| --- | --- | --- | --- | --- |
| Intercept  Baseline alcohol consumption  Age in years  Sex  Ethnicity  Smoking status  Consume fruit  Age first drink  **Alcohol-related problems**  Fighting  Parents  Friends  Sexual intercourse regretted  **Alcohol expectancy**  Feel more relaxed  More trouble with police  Forget problems  Unable to stop drinking  More friendly  Regretful activity | Change/unit (0 units)  Change/yr (14 years)  Female (male)  Asian (white)  Black (white)  Chinese (white)  Mixed (white)  Not known (white)  Other (white)  <once a month (never)  Once a month (never)  Weekly (never)  Daily (never)  <once a month (never)  Once a month (never)  Weekly (never)  Daily (never)  Change/yr (11 years)  1-2 times (none)  3-5 times (none)  6-9 times (none)  10-19 times (none)  1-2 times (none)  3-5 times (none)  6-9 times (none)  10-19 times (none)  20-39 times (none)  1-2 times (none)  3-5 times (none)  6-9 times (none)  10-19 times (none)  1-2 times (none)  3-5 times (none)  6-9 times (none)  Very likely (very unlikely)  Likely (very unlikely)  Unsure (very unlikely)  Unlikely (very unlikely)  Very likely (very unlikely)  Likely (very unlikely)  Unsure (very unlikely)  Unlikely (very unlikely)  Very likely (very unlikely)  Likely (very unlikely)  Unsure (very unlikely)  Unlikely (very unlikely)  Very likely (very unlikely)  Likely (very unlikely)  Unsure (very unlikely)  Unlikely (very unlikely)  Very likely (very unlikely)  Likely (very unlikely)  Unsure (very unlikely)  Unlikely (very unlikely)  Very likely (very unlikely)  Likely (very unlikely)  Unsure (very unlikely)  Unlikely (very unlikely) | 0.679  0.0259  0.224  -0.099  -0.0578  -0.226  -0.489  -0.198  0.395  -0.290  0.204  0.540  0.332  -0.0640  0.638  0.546  0.390  0.612  -0.0401  0.185  0.114  -0.431  0.995  0.234  -0.296  -0.101  -0.483  1.33  0.150  0.160  -0.736  -0.486  -0.0071  -0.662  -0.522  0.525  0.483  0.286  0.394  -0.329  -0.389  -0.0767  -0.109  -0.457  -0.245  -0.165  0.0348  -0.0762  0.293  0.196  0.0301  0.134  0.0513  -0.121  -0.153  0.0104  0.0445  0.0222  0.190 | 22.78  66.06  3.68  1.67  1.76  1.02  9.39  1.89  1.52  2.42  1.91  5.68  2.77  0.71  3.12  3.28  2.43 | <.001  <.001  0.05  0.13  0.13  0.40  0.002  0.11  0.18  0.05  0.13  <0.001  0.03  0.58  0.01  0.01  0.05 |
